# The Effect of Dietary Replacement of Fish Meal with Whey Protein Concentrate on the Growth Performance, Fish Health, and Immune Status of Nile Tilapia Fingerlings, *Oreochromis niloticus*

**DOI:** 10.3390/ani9121003

**Published:** 2019-11-20

**Authors:** Shimaa A. Amer, Ali Osman, Naif A. Al-Gabri, Shafika A. M. Elsayed, Ghada I. Abd El-Rahman, Mohamed Tharwat Elabbasy, Shaimaa A. A. Ahmed, Rowida E. Ibrahim

**Affiliations:** 1 Department of Nutrition & Clinical Nutrition, Faculty of Veterinary Medicine, Zagazig University, Zagazig 44511, Egypt; 2 Biochemistry Department, Faculty of Agriculture, Zagazig University, Zagazig 4451, Egypt; 3 Pathology Department, Faculty of Veterinary Medicine, Thamar University, Dahamar 1519, Yemen; 4 Department of Histology and Cytology, Faculty of Veterinary Medicine, Zagazig University, Zagazig 44511, Egypt; 5 Department of Clinical Pathology, Faculty of Veterinary Medicine, Zagazig University, Zagazig 44519, Egypt; 6 College of Public Health and Molecular Diagnostics and Personalized Therapeutics Center (CMDPT), Hail University, Hail 2440, Saudi Arabia; 7 Food Control Department, Faculty of Veterinary Medicine, Zagazig University, Zagazig 44519, Egypt; 8 Department of Fish Diseases and Management, Faculty of Veterinary Medicine, Zagazig University, Zagazig 44511, Egypt

**Keywords:** Nile tilapia, whey protein concentrate, growth, immune response, *Aeromonas hydrophila*

## Abstract

**Simple Summary:**

Although fish meal is considered the main animal protein source in fish diets, its high cost and unavailability limit its use in aquafeed. Recently, the search for other high-quality replacers of fish meal in aquatic feeds is being carried out with increased attentiveness. However, very few investigations have been performed to assess the possible use of whey protein concentrates (WPC) in Nile tilapia feeds. Five replacement percentages of fish meal with WPC (0%, 13.8%, 27.7%, 41.6%, and 55.5%) were assessed. WPC could replace the fish meal in Nile tilapia diets up to 27.7%, with improving the gut health, the total weight of survival fish, and immune status of fish challenged with *Aeromonas hydrophila*. High inclusion levels of WPC are not recommended in fish diets, since they negatively affected the intestinal and liver tissues and increased the level of cellular apoptosis, as indicated by the increased caspase 3 activity.

**Abstract:**

The present study was conducted to assess the effect of replacing fish meal with whey protein concentrate (WPC) on the growth performance, histopathological condition of organs, economic efficiency, disease resistance to intraperitoneal inoculation of *Aeromonas hydrophila*, and the immune response of *Oreochromis niloticus.* The toxicity of WPC was tested by measuring the activity of caspase 3 as an indicator of cellular apoptosis. *Oreochromis niloticus* fingerlings with average initial weight 18.65 ± 0.05 gm/fish (*n* = 225) for a 10-week feeding trial. The fish were randomly allocated to five experimental groups, having five replacement percentages of fish meal with WPC: 0%, 13.8%, 27.7%, 41.6%, and 55.5% (WPC0, WPC13.8, WPC27.7, WPC41.6, and WPC55.5); zero percentage represented the control group. The results show that the fish fed WPC had the same growth performance as the WPC0. The total weight of bacterially challenged surviving fish increased linearly and quadratically (*p* ≤ 0.05) by increasing the replacement percentage of fish meal with WPC. The growth hormone, nitric oxide, IgM, complement 3, and lysozyme activity were seen to increase significantly in WPC27.7, especially after a bacterial challenge. The phagocytic percentage and phagocytic index increased significantly in WPC27.7, WPC41.6, and WPC55.5 groups. Histopathological examination of liver sections was badly affected by high replacement in WPC41.6–55.5. The activity of caspase 3 in the immunohistochemical stained sections of the intestine was increased significantly by increasing the inclusion level of WPC. Economically, the total return of the total surviving fish after the bacterial challenge was increased significantly by fish meal replacement with WPC. It could be concluded that WPC could replace the fish meal in Nile tilapia diets up to 27.7%, with improving the gut health, the total weight of survival fish, and immune status of fish challenged with *A. hydrophila*. High inclusion levels of WPC are not recommended in fish diets, since they negatively affected the intestinal and liver tissues and increased the level of cellular apoptosis, as indicated by the increased caspase 3 activity. Further researches are recommended to evaluate the effect of fish meal replacement with WPC on the histopathological examination of the kidney and to test the capacity of serum IgM to clot the bacteria used for the challenge.

## 1. Introduction

The high worldwide demand for fish for human consumption requires a rapid growth of fish farming, which significantly reinforces the production of aquaculture feed. Fish meal is considered as a major ingredient (7–70% dry weight) of fish diets [1]. Fish meal contains ideal dietary proportions of high-quality protein and lipids essential for almost all aquaculture species; however, its high price limits its use in aquatic feeds. Additionally, the high phosphorus content in a fish meal may cause environmental troubles if overused in aquatic feeds. Thus, it is important to decrease the amount of fish meal in fish diets to reduce the excretion of excess phosphorus [2,3,4]. Currently, the search for other high-quality replacers of fish meal in aquatic feeds is being carried out with increased attentiveness. However, very few investigations have been performed to assess the possible use of whey protein concentrates in Nile tilapia feeds.

Whey, a by-product of cheese making or casein in dairy production, is valuable because it contains soluble proteins, high levels of essential amino acids, vitamin B, lactose, and salts [5]. Although whey has high biological value, its use in nature is restricted, since its components are delicate and more diluted. However, whey can be concentrated using certain membrane separation technologies to produce whey protein concentrate (WPC), which contains 35–80% proteins [6]. Whey contains a variety of immunomodulatory compounds, like major proteins (β-lactoglobulin and α-lactalbumin), minor proteins (lactoferrin and lactoperoxidase), and tissue growth factors (such as TGFh) [7,8,9,10,11], while WPCs contain combinations of these constituents [12]. Whey proteins have distinctive properties [13] that give them more importance in nutrition; they display physical, chemical, functional, physiological, and technological features moreover beneficial for food processing [14]. Whey recovered proteins have a role in industrial purposes. These proteins, either concentrated or isolated, and their derived biological active peptides were given more interest for their use in pharmaceutical and food manufacturing, and this consequently prevents the pollution problems related to the disposal of whey [15].

Whey protein contains a high number of essential amino acids that play an important role in protein synthesis and carbohydrate metabolism required for supplying energy [16]. Traces of organic acids, such as lactic acid and citric acid, are also found in whey protein [17]. Research studies on the application of WPC as an alternative animal protein source in fish diets are limited. Abdel-Tawwab and Abbass [18] investigated the effect of substitution of fish meal with dried whey meal by 25, 50, 75, or 100% in Nile tilapia, *Oreochromis niloticus,* and they found no significant effect of this substitution on the growth performance, nutrient utilization, and the whole body composition of fish, except for the lipid content of fish body, which increased in 100% fish meal replacement compared with the 0%. The immune-modulatory effect of whey protein concentrate was tested in the present study by challenging Nile tilapia fingerlings by *Aeromonas hydrophila*. *Aeromonas hydrophila* is an important pathogen of different fish species [19], causing huge economic losses in aquaculture [20].

Therefore, this study was done to investigate the effect of replacing the fish meal with whey protein concentrate (WPC) in the diets of Nile tilapia fingerlings. In this regard, the growth performance, body composition, fish health, and immune response of the fish challenged with *A. hydrophila* were studied after a 10-week feeding period, along with histopathological analysis of some organs of Nile tilapia fingerlings. The toxicity of WPC was tested by measuring the activity of caspase 3 as indicator of cell apoptosis.

## 2. Materials and Methods

### 2.1. Fish and Experimental Design

This study was approved by the Animal Research Ethics Committee of Zagazig University and the experimental procedures were done following the NIH general guidelines for the Care and Use of Laboratory Animals in scientific investigations. Two hundred and twenty-five healthy Nile tilapia fingerlings (*Oreochromis niloticus*) with initial weight 18.65 ± 0.05 gm/fish were obtained from local Fish Hatchery, Sharkia Governorate, Egypt. The fish were stocked in 15 glass aquaria (80 × 40 × 30 cm) and allotted randomly into five experimental groups with three replicates each (15 fish/replicate). Each glass aquarium represents a replicate. The experimental groups consisted of five different replacement percentages of fish meal with whey protein concentrate (WPC0, WPC13.8, WPC27.7, WPC41.6, and WPC55.5) with 0%representing the control group. The water parameters were monitored [21] and maintained within the ranges proposed during the experiment (pH: 7.2 ± 0.5; ammonia: 0.02 ± 0.001 mg/L; nitrite: 0.017± 0.001 mg/L; water temperature: 24 ± 2 °C; and photoperiod: 12:12 light/dark). Whole water exchange was performed twice a week.

### 2.2. Preparation and Characterization of Whey Protein Concentrate and Experimental Diet Formulation

Whey was prepared from buffalo milk acquired from a market in Zagazig, Egypt, using rennet coagulation according to the method of Ha et al. [22], as described in Kishawy et al. [23,24]. The whey was used to prepare WPC, according to Nishanthi et al. [25].

Sodium dodecyl sulfate–polyacrylamide gel electrophoresis “SDS-PAGE” of WPC was applied according to an earlier report [26]. Briefly, 50 mg of whey protein was mixed with 1 mL of loading buffer “0.3 M Tris-HCl (pH 6.8), 5% SDS, 50% glycerol and bromophenol blue traces“, heated at 95 °C for 5 min, and 10 µL of this mixture was loaded per well [27]. For Fourier transform infrared spectroscopy (FTIR analysis), WPC was prepared with potassium bromide [28]. Infrared spectra were measured with an FTIR spectrometer (NICOLET NEXUS 470, DTGS, Thermo Scientific, Waltham, MA, USA) at 25 °C. Whey protein concentrate was analyzed according to [29], where DM = 92.4%, CP = 38%, fat = 0.02%.

Iso-energetic and iso-protein diets (Table 1) were formulated according to the standard measures [30], considering the dry matter, crude protein, ether extract, crude fiber, and ash. The digestible energy (DE) contents of the experimental diets were calculated from the values of protein 3.5 kcal gm^−1^, fat 8.1 kcal gm^–1^, NFE 2.5 kcal gm^–1^ [31]. Dietetic feed ingredients were prepared in the form of pellets (2 mm). Before the beginning of the trial, an adaptation period of 14 days was provided to the fish during which the Nile tilapia fingerlings were fed the control diet. Hand feeding of fish was done two times per day, for 10 weeks, until satiation was reached. Proximate chemical analysis of fish and diets were done according to [29].

### 2.3. Survival Percentage and Growth Performance Parameters

The fish were weighed and the feed intake was recorded at the start and end of the trial period. Total weight gain, average daily gain, specific growth rate, and feed conversion ratio were calculated [32]. The protein efficiency ratio (PER) was determined according to an earlier report [33].

Various parameters of fish growth were as follows:

Total gain (g fish^–1^) = (WT − WI), where WT is the final weight of fish in grams and WI is the initial weight of fish in grams.

The average daily gain (ADG) (g fish^–1^ day^–1^) = total gain/experimental days.

The specific growth rate (SGR) (% day^–1^) = 100 × (ln WT − ln WI)/duration/day.

The feed conversion ratio (FCR) = total feed intake (g)/total gain (g).

The protein efficiency ratio (PER) = total gain (g)/protein intake (g).

Survival percentage = (number of fish in each group remaining after the 10-week feeding period/initial number of fish) × 100. The total weight of surviving fish was recorded after bacterial challenge.

### 2.4. Bacterial Challenge Test

At the end of the trial, all experimental groups were intraperitoneally inoculated with pathogenic A. hydrophila at a dose of 0.1 mL cell suspension containing 1.5 × 107 cells/mL by using McFarland standard tubes. The isolate was previously isolated from moribund fish at the Department of Fish Diseases and Management, Faculty of Veterinary Medicine, Zagazig University and confirmed to be pathogenic for O. niloticus. A. hydrophila was identified by conventional biochemical tests and VITEK 2-C15 automated system for bacterial identification (BioMérieux, Marcy-l'Étoile, France) according to the manufacturer’s instructions, as described by [34,35] at Microbiology and Immunology Department, National Research Centre (NRC), Dokki, Giza, Egypt. Fish mortalities and clinical signs were observed for two weeks, according to [36].

### 2.5. Sampling

At the end of feeding trial and 14 days post-fish experimental challenge, whole blood samples with Ethylenediaminetetraaceticacid (EDTA) were taken for measurement of phagocytosis. Additionally, blood samples without anticoagulant were collected and left to clot at room temperature or in the refrigerator for 1 h, followed by centrifugation at 3000 rpm for 15 min. The clear supernatant (serum) was transferred into dry, sterile, labeled, and stoppered vials to be used for determination of liver and kidney function tests, immunological parameters, and antioxidant activity. All samples were taken in triplicate.

### 2.6. Fish Whole Body Composition

At the end of the experimental period, six fish per group were sampled and frozen at −20 °C till analyzed for the determination of whole fish body composition. The frozen whole fish were thawed, dried in the hot air oven, blended, and analyzed for determination of moisture, crude protein, ether extract, and ash content according to Official Analytical Chemists protocol [29]. Moisture content was estimated by drying the samples to constant weight at 85 °C in a drying oven (GCA, model 18EM, Precision Scientific Group, Chicago, IL, USA). Nitrogen content was measured using a micro Kjeldahl apparatus (Labconco, Labconco Corporation, Kansas, MO, USA), and crude protein was estimated by multiplying nitrogen content by 6.25. Lipid content was determined by ether extraction in a multiunit extraction Soxhlet apparatus (Lab-Line Instruments, Inc., Melrose Park, IL, USA) for 16 h, and total ash was determined by combusting dry samples in a muffle furnace (Thermolyne Corporation, Dubuque, IA, USA) at 550 °C for 6 h.

Sensory characteristics and general acceptance of fish samples were assessed by a six-member committee. The assessment was conducted on a 10-point scale from each sample and included the overall appearance, smell, and texture of the fish [37].

### 2.7. Blood Biochemical Parameters

#### 2.7.1. Phagocytic Capacity

The white blood cells were separated from peripheral blood of the tested fish belonging to different experimental groups. Heat-inactivated *Candida albicans*, which was isolated and identified at the Department of Bacteriology, Mycology, and Immunity, Faculty of Veterinary Medicine, Zagazig University, was used to determine the phagocytic capacity of the phagocytic cells in each experimental group [38]. The slides were air-dried and stained sequentially with Leishman’s stain, and viewed under oil immersion at 100x. Approximately 100 cells were counted from random fields of view and the numbers of phagocytic cells were recorded.

The percent phagocytosis and the phagocytic index were calculated according to the formula described by [39], after counting at least 100 phagocytic cells either phagocytizing or not for the calculation of the percent phagocytosis, and by counting at least 100 bacteria that were phagocytized by certain number of phagocytic cells/macrophages for the phagocytic index calculation.

Phagocytic index = Total no. of phagocytized bacteria/No. of phagocytic cells phagocytizing bacteria

% Phagocytosis = (No. of phagocytic cell phagocytizing bacteria/Total no. of phagocytic cells counted) × 100

#### 2.7.2. The Blood Levels of IgM, Complement 3, and Lysozyme Activity

Serum IgM was measured using a fish-specific ELISA kits with CAT. NO. MBS282651 (sensitivity: 1 μg/mL, control of the reaction: 1.56–100 µg/mL), following the instructions of the manufacturer (MyBioSource Company, San Diego, USA). Serum complement 3 was measured using a fish-specific ELISA kits with CAT. NO. MBS005953 (sensitivity: 1 µg/mL, control of the reaction: 31.2–1000 µg/mL), following the instructions of the manufacturer (MyBioSource Company, Diego, USA). Lysozyme activity was measured using a Fish Lysozyme ELISA kits with CAT. NO. MBS099538 (sensitivity: 1.0 µg/mL, control of the reaction: 1.25–40 µg/mL), following the instructions of the manufacturer (MyBioSource Company, Diego, USA).

#### 2.7.3. Liver and Kidney Function Tests

Liver function tests (ALT and AST) and kidney function tests (urea and creatinine) were performed using the kits by Spinreact (Esteve De Bas, Girona, Spain), according to the methods of Murray [40], Burtis and Ashwood [41], Kaplan [42], and Fossati, et al. [43], respectively.

#### 2.7.4. The Blood Levels of Growth Hormone and Nitric Oxide

The level of Nitric oxide (NO) was measured according to [44]. Meanwhile, a Fish Growth Hormone ELISA kit of MyBiosource Co. with CAT. NO. MBS044656 (sensitivity: 1.0 ng/mL, control of the reaction: is 1.56–50 ng/mL) was used for growth hormone (GH) determination, following the instructions of the enclosed pamphlets.

### 2.8. Histology and Morphometric Methods

The tissue specimens of intestine and liver were collected from each group at the end of feeding trial and 14 days post-fish experimental challenge, and fixed in 10% neutral buffered formalin. The specimens were dehydrated in ascending grades of ethyl alcohol (70–100%), cleared in xylene, and embedded in paraffin. Tissue sections of 5 µm thicknesses were prepared with the help of microtome (Leica^®^, Wetzlar, Germany), and stained with hematoxylin and eosin (H&E) and periodic acid Schiff (PAS). Slides were examined for morphometric analysis and photographed using AmScope digital camera-attached Ceti England microscope for histopathological examination [45]. Morphometric analyses were done using 20 images per animal captured at 40x and 400x for villus height and width, crypt depth, lymphocytes, and Goblet cells counting using AmscopeToupView 3.7 software (AmScope, California, USA).

### 2.9. Immunohistochemistry (IHC)

Immunohistochemical staining for Caspase 3 was done according to the ABC technique described by [46]. Briefly, paraffin-embedded intestinal sections of 3 μm thickness were deparaffinized in xylene and then rehydrated in ethyl alcohol. The sections were incubated for 2 h with 5% bovine serum albumin in Tris-buffered saline (TBS) for blocking of the non-specific binding sites. The sections were incubated with primary antibodies rabbit anti-p53 antibody (catalog ab31333) Abcam^®^ (Cambridge, MA, USA) at a dilution of 1:300 with 5% BSA in TBS and incubated overnight at 4 °C. Post-incubation, the slides were washed 3 times by TBS, then incubated with secondary antibody goat antirabbit conjugated to horseradish peroxidase (catalog ab6721) Abcam^®^ (Cambridge, MA, USA) for 1 h at room temperature. Sections were washed by TBS then incubated for 7 min in 0.02% diaminobenzidine (DAB) containing 0.01% hydrogen peroxide. Sections were counterstained by hematoxylin and the slides were visualized under the microscope. Immunostaining positive area percentages were measured by ImageJ software in 5 sections in each group at high magnification, according to [47].

### 2.10. Economic Efficiency

Collective efficiency measures were calculated according to El-Telbany and Atallah [48] and Dunning and Daniels [49]. It includes total return, total costs, variable costs, and net profit. Performance index (PI) was calculated according to North and Bell [50].

### 2.11. Statistical Analysis

All data are expressed as a mean ± standard error (SE). All data were verified for normality after transformation (ASIN). ANOVA test was applied based on polynomial orthogonal contrasts. Linear and quadratic regression equations were calculated using SPSS Version 17 for Windows (SPSS Inc., Chicago, IL, USA) to determine the effect of fish meal substitution with WPC in *Oreochromis niloticus* diet on the growth, health, economic efficiency, and immune response of fish challenged with *A. hydrophila*, and histopathological examination of specific organs. The regressions were considered significant when *p* ≤ 0.05. Post-hoc Tukey’s test was used to determine differences among means. Statements of statistical significance are based on *p* ≤ 0.05, unless otherwise stated.

## 3. Results

### 3.1. Characterization of Whey Protein Concentrate

**Figure 1:**
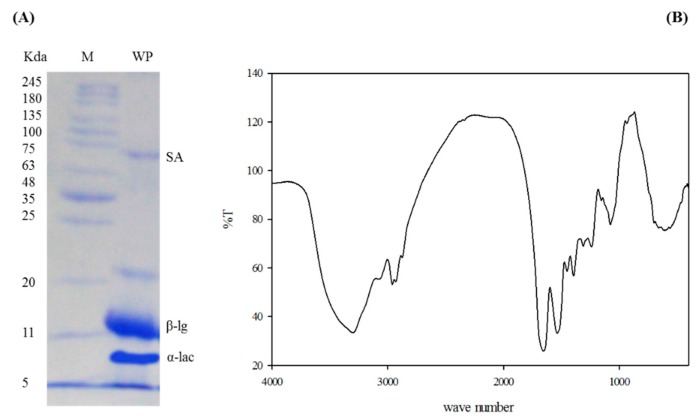
SDS-PAGE of whey protein concentrate (**A**). M: Protein marker; WP: Whey protein; α-lac: Alpha lacto-albumin; β-lg: Beta lacto-globulin; and FTIR spectra of whey protein concentrate (**B**).

The molecular weights of WPC were determined by comparing with protein markers of molecular weights arranged from 245 to 5 kDa. SDS-electrophoretic profile of WPC presented three major fractions that were identical to serum albumin (SA), β-lactoglobulin (β-LG), and α-Lacto-albumin (α-lac) (Figure 1A). In the calculated spectral range of 4000 to 400 cm^−1^, there were considerable peaks that matched the various molecular bonds of WPC components. The most pertinent peaks that were recorded between 3500 and 3000 cm^–1^ and between 1800 and 1000 cm^−1^ are shown in Figure 1B.

### 3.2. Survival Percentage and Growth Performance

As shown in Table 2, the replacement of fish meal with WPC did not affect (*p* > 0.05) the body weight, weight gain, feed conversion ratio, daily weight gain, or specific growth rate, neither linearly nor quadratically, when compared to WPC0. The survival percentage and the total weight of the surviving fish after bacterial challenge increased linearly and quadratically (*p* ≤ 0.05) by increasing the replacement percentage when compared with WPC0 group. The survival percentages for WPC0, WPC13.8, WPC27.7 to WPC41.6, and WPC55.5 were 66.66%, 88.88%, 91.11%, 95.55%, and 97.77%, respectively.

Samples of examined fish were viewed as worthy for human utilization until the sensory score arrived at 4. All analyzed gatherings at zero time were fresh and had high scores extending from 8.7 to 9.3.

### 3.3. Fish Whole body Proximate Composition

Table 2 also highlights the effect of substitution of fish meal with the graded levels of WPC on the whole body composition of fish. The results indicate that the dry matter and crude protein content in the fish body in WPC13.8 to WPC55.5 did not differ significantly (*p* > 0.05) from WPC0. The body fat was increased linearly (*p* = 0.02) in WPC27.7. The body ash was increased linearly (*p* = 0.01) in WPC41.6 and WPC55.5.

### 3.4. Clinical Signs and Fish Behavior

During the experimental period and before the bacterial challenge, fish in all experimental groups showed normal clinical signs and behavior. However, after the bacterial challenge, the fish group fed on WPC0 showed hemorrhage and erythema on the skin, anorexia, and sluggish swimming. The fish group fed on WPC13.8 showed slight hemorrhage and erythema on the skin, decreased feed intake, and decreased swimming activity. The fish groups fed on WPC27.7 to WPC41.6 showed the same clinical signs with mild fin rot, normal appetite, and swimming activity, while the fish group fed on WPC55.5 showed slight darkness of body, along with normal appetite and swimming activity.

### 3.5. Growth Hormone and Nitric Oxide (NO) Levels

At the end of the experiment, the level of the growth hormone increased linearly and quadratically (*p* ≤ 0.05) by increasing the replacement percent of fish meal with WPC (Table 3). Whereas, after bacterial challenge, the level was found to improve quadratically (*p* = 0.00) in the WPC27.7 group (Table 4). At the end of the experiment, the level of nitric oxide was increased linearly (*p* = 0.02) by increasing the percent of WPC inclusion (Table 3), while after the bacterial challenge, it was increased quadratically (*p* = 0.00) in the WPC27.7 group (Table 4).

### 3.6. Levels of IgM, Complement 3, and Lysozyme Activity

Before the bacterial challenge test, the level of IgM was not significantly affected (*p* > 0.05) by WPC inclusion. The lysozyme activity and the level of complement 3 were seen to increase linearly (*p* ≤ 0.05) with increasing WPC percentage (Table 3). After the bacterial challenge, the lysozyme activity and the levels of complement 3 and IgM were increased quadratically (*p* ≤ 0.05) in the WPC27.7 group compared to the WPC0 group (Table 4).

### 3.7. Liver Function Tests

At the end of the experiment, the level of ALT increased quadratically (*p* = 0.00) in the WPC55.5 group in comparison to the WPC0 group. In comparison to the WPC0 group, the level of AST was increased both linearly and quadratically (*p* = 0.00) in the WPC55.5 group (Table 3). Post-bacterial challenge, the level of ALT was seen to decrease linearly and quadratically (*p* = 0.00) due to WPC inclusion. The level of AST was found to increase both linearly and quadratically (*p* = 0.00) in the WPC55.5 group (Table 4).

### 3.8. Kidney Function Tests

At the end of the experiment, the levels of urea and creatinine increased significantly (*p* = 0.00) in the group fed on a diet with WPC41.6, while the same decreased significantly (*p* = 0.00) in the WPC55.5 group compared to the WPC0 group (Table 3). Post-bacterial challenge, the level of urea significantly increased (*p* ≤ 0.05) in the WPC13.8 to WPC41.6 groups, with the highest value at WPC27.7, while it significantly decreased (*p* ≤ 0.05) in the WPC55.5 group, in comparison to the WPC0 group. The level of creatinine was linearly and quadratically decreased (*p* ≤ 0.05) due to WPC inclusion, wherein the lowest value was reported in the WPC41.6 group (Table 4).

### 3.9. Phagocytic Percentage and Phagocytic Index

At the end of the experiment, the phagocytic percentage was higher (*p* ≤ 0.05) in the WPC27.7 to WPC41.6 groups in comparison with the WPC0 group (Table 3). While after the bacterial challenge, the phagocytic percentage was increased (*p* ≤ 0.05) by increasing the level of WPC inclusion. The phagocytic index was improved (*p* ≤ 0.05) in the WPC27.7 to WPC55.5 groups in comparison with the WPC0 group, while the highest value was reported in the WPC27.7 group (Table 4).

### 3.10. Histological Findings

**Figure 2:**
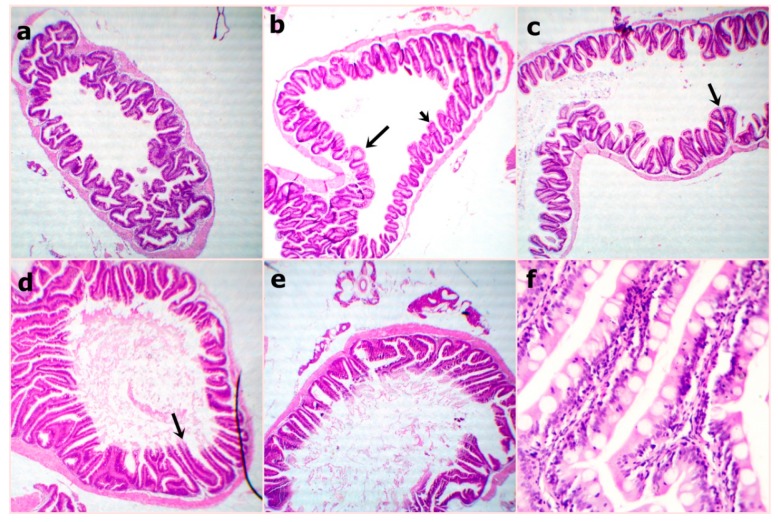
Representative photomicrograph of H&E stained sections from fish intestine at the end of the experiment showing (**a**) WPC0–reduced villus height and width at 40x; (**b**) WPC13.8—distinctly arranged tall villi at 40x; (**c**) WPC27.7—marked tall and branched villi with some broad tips (arrow) at 40x; (**d**) WPC41.6—marked very tall and thin villi (arrow) at 40x; (**e**,**f**) WPC55.5—clearly tall and thin villi with partially damaged tips (arrow) with reduced goblet cell count and inter-epithelium lymphocytic infiltration (arrow) at low 40x and high magnification 400x.

**Figure 3:**
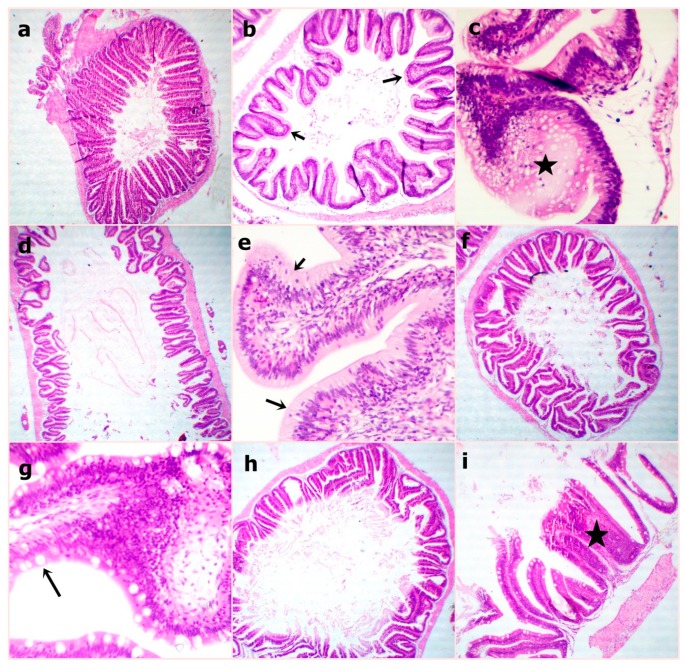
Representative photomicrograph of H&E stained sections from fish intestine after bacterial challenge showing (**a**) WPC0—normal intestinal villous structures at 40x; (**b**,**c**) WPC13.8—the apparently tall villous with numerous broad tips (arrows) and goblet cell metaplasia (star) at low 40x and high magnification 400x; (**d**,**e**) WPC27.7—apparently short and thick villous with increased intraepithelial lymphocytic infiltrations (arrows) at low 40x and high magnification 400x; (**f**,**g**) WPC41.6—nearly normal intestinal villous height and limited goblet cell metaplasia (arrow) at low 40x and high magnification 400x; (**h**,**I**) WPC55.5—apparently normal intestinal villous height with marked fusion (star) at low 40x and high magnification 100x.

At the end of the experiment, the examined sections from fish intestine revealed the lowest villus height and width in the WPC0 group. The sections from the WPC13.8 group revealed tall and branched villi with some broad tips. The sections from WPC27.7 showed distinct, very tall, and thin villi. Clear, tall, and thin villi were observed in the sections from the WPC41.6. to WPC55.5 groups with limited goblet cell count and inter-epithelium lymphocytic infiltrations (Figure 2). The fish intestinal sections exposed to bacterial challenge showed normal intestinal villus structures in the WPC0 group. The WPC13.8 group revealed tall villi with numerous broad tips and goblet cell metaplasia. The sections from the WPC27.7 group at the lowest and highest magnification showed widening of lacteal with the limitation of goblet cells. The WPC41.6 group displayed near normal intestinal villus height and limited goblet cell metaplasia. The WPC55.5 group showed normal intestinal villus height with marked fusion (Figure 3).

**Figure 4:**
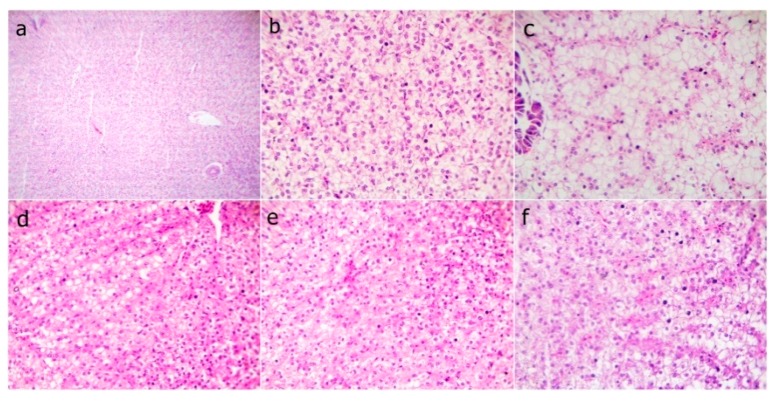
Representative photomicrograph of H&E stained sections from fish liver before the bacterial challenge showing (**a**) WPC0—at low 40x and (**b**) WPC0—high magnification 400x of the normal hepatic structures; (**c**) WPC13.8—mild fatty hepatocytes at 400x; (**d**) WPC27.7 and (**e**) WPC41.6—markedly narrowed sinusoids with slightly congested sinusoids at 400x; (**f**) WPC55.5—normal hepatocytes and slightly congested sinusoids at 400x.

**Figure 5:**
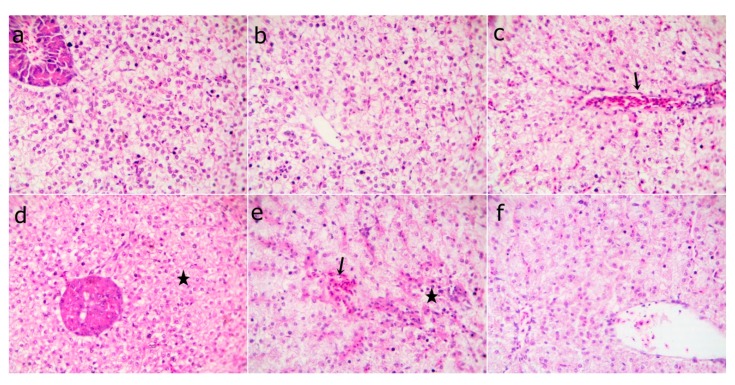
Representative photomicrograph of the H&E stained sections from fish liver after bacterial challenge showing (**a**) WPC0—normal histomorphological structures at 400x; (**b**) WPC13.8—normal central vein and hepatocytes at 400x; (**c**, WPC27.7) markedly congested hepatic blood vessels (arrow) at 400x; (**d**) WPC41.6—degenerated hepatocytes with disappeared sinusoids (star) at 400x; (**e**) WPC55.5—mildly congested sinusoids (arrow) and degenerated hepatocytes (star) at 400x; (**f**) WPC55.5—nearly normal hepatic structures at 400x.

The fish liver sections that were examined at the end of the experiment showed normal hepatic structures in the WPC0 group. The WPC13.8 group with mild fatty hepatocytes, the WPC27.7 to WPC41.6 groups having marked narrowing sinusoids with slightly congested sinusoids, and the WPC55.5 group with normal hepatocytes and slightly congested sinusoids (Figure 4) were observed. The liver sections from fish exposed to the bacterial challenge showed normal histomorphological structures in the WPC0 group. The WPC13.8 group revealed normal central vein and hepatocytes. The WPC27.7 group showed marked congested hepatic blood vessels and the WPC41.6 group had degenerated hepatocytes with the disappearance of sinusoids. The WPC55.5 group displayed mildly congested sinusoids, degenerated hepatocytes, and nearly normal hepatic structures (Figure 5).

**Figure 6:**
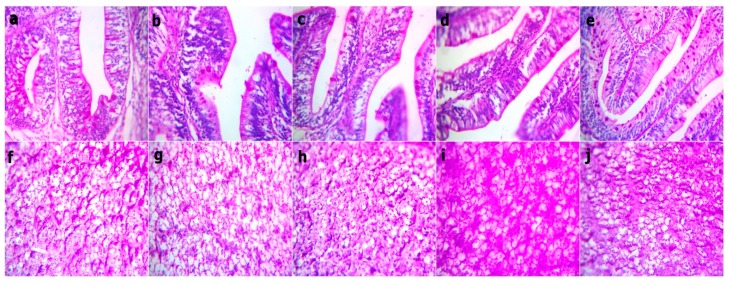
Representative photomicrograph of the Periodic Acid Schiff stained sections at magnification 400x from the fish intestine and liver before bacterial challenge showing the red stainable intestinal goblet cells and mucus within the limit and no difference between all groups. Red granular stainable materials (glycogen) distributed in the hepatocyte cytoplasms are within the normal limit, but they are mildly increased in the WPC41.6 and WPC55.5 liver sections. (**a**,**f**) WPC0, (**b**,**g**) WPC13.8, (**c**,**h**) WPC27.7, (**d**,**i**) WPC41.6, (**e**,**j**) WPC55.5.

**Figure 7:**
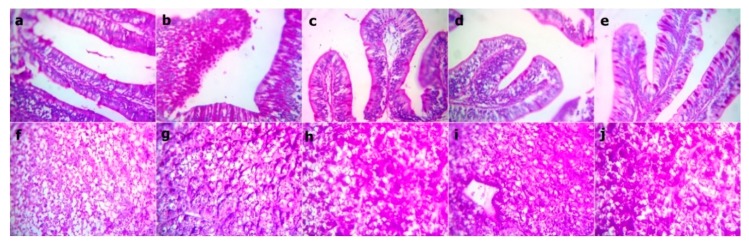
Representative photomicrograph of the Periodic Acid Schiff stained sections at magnification 400x from fish intestine and liver after bacterial challenge showing an increase in the red stainable intestinal goblet cells and mucous in WPC13.8 and within the limit no difference between the other groups. While, mild to moderate increase in the red granular stainable materials (glycogen) distributed in the hepatocyte cytoplasms in the WPC41.6 and WPC55.5 groups, while they are within the normal limit in the other liver sections. (**a**,**f**, WPC0, (**b**,**g**) WPC13.8, (**c**,**h**) WPC27.7, (**d**,**i**) WPC41.6, (**e**,**j**) WPC55.5.

The Periodic Acid Schiff (PAS) stained sections from fish intestine and liver before bacterial challenge revealed red stainable intestinal goblet cells and mucus within the normal limit and no difference between groups, while there was a mild increase in the red granular stainable materials (glycogen) distributed in the hepatocyte cytoplasms in both the WPC41.6 and WPC55.5 groups (Figure 6). After the bacterial challenge, there was an increase in the red stainable goblet cells and mucous in WPC13.8 only, and no difference between the other groups. The liver sections revealed a mild to moderate increase in both WPC41.6 and WPC55.5, besides within the normal limit in the other groups (Figure 7).

### 3.11. Morphometric Measures of the Intestine

The morphometric measures of the intestine are shown in Table 5. Villus height was increased linearly (*p* = 0.05) in the WPC41.6 and WPC55.5 groups, unlike in the WPC0 group. In comparison with the WPC0 group, the goblet cells were not significantly affected (*p* > 0.05) by WPC inclusion before the bacterial challenge, but they were linearly increased (*p* = 0.00) after bacterial challenge in the WPC13.8 group. It was found that the villus width was not significantly affected (*p* > 0.05) by WPC inclusion. Crypt depth decreased linearly (*p* = 0.001) by increasing the percentage of WPC inclusion before the bacterial challenge, in comparison with the WPC0 group, while it was not significantly affected (*p* > 0.05) after bacterial challenge due to the WPC inclusion. The inter-epithelium lymphocytic infiltrations were decreased linearly and quadratically (*p* = 0.00) in the WPC41.6 and WPC55.5 groups before the bacterial challenge, while their count was decreased linearly and quadratically (*p* = 0.01) only in the WPC55.5 group after the bacterial challenge (Table 5).

### 3.12. Immunohistochemistry Results

**Figure 8:**
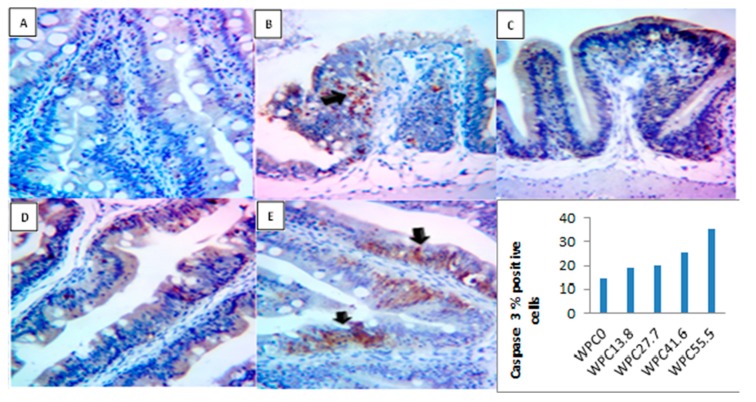
Representative photomicrograph of the Caspase 3 IHC stained sections and quantification analysis for area % of Caspase 3 immunopositive cells at magnification 400x from the fish intestine before bacterial challenge showing: (**A**) Limited brownish stained enterocytes in WPC0; (**B**,**C**) slight brown stained deposits in the numerous enterocytes in both WPC13.8 and WPC27.7; (**D**) marked stained enterocytes and numerous cells in lamina propria (arrow) in WPC41.6; (**E**) marked to intense brownish stained enterocytes especially in the basal villi (arrows) in WPC55.5.

As shown in Figure 8 the activity of caspase 3 in the immunohistochemical sections of the intestine at the end of the experimental period was increased linearly and quadratically (*p* = 0.00) with increasing the level of replacement of fish meal with WPC, where the highest level was reported in the WPC41.6–55.5 groups. Also, this figure showed limited brownish stained enterocytes in WPC0, slight brown stained deposits in the numerous enterocytes in both WPC13.8 and WPC27.7, marked stained enterocytes and numerous cells in lamina propria in WPC41.6, and marked to intense brownish stained enterocytes especially in the basal villi in WPC55.5.

### 3.13. Economic Efficiency of the Experimental Diets

Table 6 highlights the effects of fish meal replacement with WPC on the economic efficiency of the experimental diets. The results show that the total return, net profit, feed cost, total cost, performance index, economic efficiency, and feed cost/kilogram gain were not linearly or quadratically affected (*p* > 0.05) by WPC inclusion. The total return of the total fish surviving after the bacterial challenge was increased linearly (*p* = 0.005) and quadratically (*p* = 0.01) by fish meal substitution with WPC.

## 4. Discussion

Three major fractions identical to SA, β-LG, and α-LAC were present in WPC. The same results were reported earlier [51,52]. In the FTIR spectra, there were two main peaks for Amide I at 1640 cm^−1^ and Amide II at 1550 cm^−1^, related to peptide bonds [53].

Whey protein concentrate is considered to be a protein supplement which contains 35–80% proteins [6] and an immune potentiating agent [12], which makes it desirable for fish meal replacement. There is a paucity of research studies that evaluate the use of whey protein concentrates as a replacement of fish meal in fish diets. In this study, the inclusion of whey protein at different levels in the diets of Nile tilapia fingerlings as a partial replacement of fish meal results in the same growth performance as the control group that received zero WPC (100% fish meal diet). This could be attributed to the high content of essential amino acids required for protein synthesis and energy [16]. This may also be attributed to the role of WPC in increasing the growth hormone, in addition to its role in improving the gut health, increasing the intestinal absorptive surface, and increasing the number of goblet cells that were observed in the results of the present study. These results are similar to the results of [18], who reported no statically difference in the growth, feed efficiency, PER, protein, and energy utilization by using dry whey meal as fish meal substitution in the diets of *Oreochromis niloticus* fingerlings by 25%, 50%, 75%, and 100%. Therefore, whey proteins have a major nutritional importance for growth and cellular repair, as they supply energy and essential amino acids [15].

Concerning the proximate composition of whole fish, the results also showed that the body ash increased at higher replacement percentage of WPC (WPC41.6–55.5). The high bioavailability of minerals in WPC, especially calcium and phosphorus, explain the increased ash content in the fish body. Dixon and Perkins [54] suggested that co-precipitation during bone salt composition resulted in a local increase in citric acid a constituent of WPC in bone matrix. These results confirm our previous reports, suggesting that whey protein can improve growth performance and gut health, and also increase the ash content in the whole body composition [23]. The results of [18] showed no significant effect of fish meal substitution with dry whey meal up to 100% on the body composition from moisture, crude protein, and total ash, while the body fat content was significantly increased in 100% dry whey meal group compared to the control group.

Samples of examined fish were viewed as worthy for human utilization until the sensory score arrived at 4 [55]. All analyzed gatherings at zero time were in a fresh way and had high scores extending from 8.7 to 9.3; this demonstrates all examined samples were of superb quality [37].

In this study, the immunomodulatory effect of WPC was confirmed with the help of bacterial challenge. After challenging the fish with *A. hydrophila*, severe clinical signs were recorded in the WPC0 group. These were hemorrhages, erythema, sluggish swimming, and poor reflexes, with a high mortality rate, septicemia, and hemorrhage in all internal organs, spleen, liver, kidney, and gills. By increasing the replacement percentage of fish meal with WPC, the clinical signs reduced gradually. The mortality rate was seen to decrease due to WPC inclusion. The total weight of the surviving fish that had been exposed to the bacterial challenge was increased in the WPC supplemented groups, and this has economic importance, where the total return of the total fish surviving after the bacterial challenge was increased significantly by fish meal replacement with WPC. This was achieved by the increase in the phagocytic percentage and phagocytic index, improved blood immunological parameters, such as IgM, complement 3, nitric oxide, and an increase in the lysozyme activity in the WPC groups. We recommend further studies testing the capacity of serum IgM to clot the bacteria used for the challenge. Our results explain the role of WPC in potentiating the innate immunity by increasing the proliferation and expansion of splenocytes and enhancing the innate leukocytes responses through phagocytosis [10,56,57]. Whey proteins potentiate immune responses more significantly than casein or soy protein-containing diets [15]. Ingestion of α-lactalbumin, β-lactoglobulin (β-LG), the bioactive compounds of whey protein, resulted in antimicrobial and antiviral actions, and stimulation of the immune system [58,59,60,61,62,63]. Also, they contain bovine serum albumin (BSA), immunoglobulins, lactoferrin, and glycomacropeptide, which improve humoral immunity [10,64,65,66]. β-Lactoglobulin, the main protein in WPC, is considered as a precursor of bioactive peptides that carries immunomodulatory function [67]. The high biological value of WPC as a good digestible protein supplement, with high levels of sulfur-containing amino acids, tryptophan, could be the reason why WPC is an immune-potentiating dietary protein supplement [68,69,70,71]. Therefore, progresses in dietary biochemistry and biomedical research have actually revealed the complex relations between nutrition and disease, suggesting that dietary proteins and peptides that have arisen during digestion (or from in vitro protein degradation) may play significant roles in preventing or treating related diseases with malnutrition, pathogens, and injuries [72].

Chi et al. [73] reported improved lysosomal and phagocytic activity with the inclusion of whey in Barramundi, *Lates calcarifer,* diet. The increase in the phagocytic percentage, phagocytic index, and nitric oxide level by WPC inclusion was attributed to the stimulation of the macrophage [74], [75]. Nitric oxide is concerned with vasodilatation, neurotransmission, inhibition of platelet aggregation, the transmission of inflammatory reactions, and direct destruction of invading microbes [76].

Moreover, some whey protein-derived peptides have favorable health effects on the digestive, nervous, or immune systems, which include antimicrobial properties, and antioxidant activities, improvement of mineral absorption, and consequently, immune-modulatory effects. The mechanisms of the physiological roles of these bioactive peptides involve action upon certain receptors, enzyme inhibitors, intestinal absorption regulation, and display antioxidant or antimicrobial activities [15]. Whey proteins have antioxidant activity, possibly through radicals scavenging through Tyrosine and Cysteine amino acid residues that rely mainly on the transport mechanisms of the hydrogen atom or the single electron associated with the proton [77,78].

Whey proteins play an important role in improving growth performance, potentiating immune response, increasing resistance against bacterial infection, increasing survival rate, and improving gut health. Nonetheless, their inclusion at high levels in the fish meal (WPC41.6–55.5) in Nile tilapia diets, for a long period, resulted in some alterations in the liver tissues, such as the microscopic appearance of some destructed and swollen hepatocytes, congested hepatic blood vessels, and a mild increase in the red granular stainable materials (glycogen) distributed in the hepatocyte cytoplasms. These alterations were reflected in the blood levels of AST, ALT, urea, and creatinine. So, we also recommend further researches to evaluate the effect of fish meal replacement with WPC on the histopathological examination of the kidney. AST and ALT are the common diagnostic indicators of liver diseases, and their increased serum levels indicate hepatic damage [79]. A decline in the liver function also leads to decreased creatine production and lower serum creatinine levels because of decreased creatine storage and less conversion of creatine to creatinine [80]. Whey proteins have an essential role in carbohydrate metabolism by supplying the required energy [81]. Liver glycogen stores are exhausted after rigorous physical activity [82]. Whey protein is considered to be a useful supplement for tissue and liver glycogen during work and exercise [83]. Moreover, the toxicity of WPC was tested by measuring the activity of caspase 3 as an indicator of cellular apoptosis [84]. The results of the present study reveal a significant increase in the caspase 3 activity in the highest inclusion levels of WPC (WPC41.6–55.5). Apoptosis is an extremely controlled and organized cellular process through the removal of cells from the tissue by the activation of specific death-signaling pathways. These pathways are characterized by reflective and distinct alterations in cellular construction, resulting in self-damage; also, it arises as part of regular growth and aging. However, different physiological and patho-physiological stimuli, including the damage stimuli from environmental pollutants, also require the apoptosis process [85]. There are two major pathways of apoptosis; the intrinsic or mitochondrial pathway, and the extrinsic or the death receptor pathway [86,87,88]. The active component of the extrinsic pathway is a cataract of cysteinyl aspartic acid-specific proteases known as caspases. The caspases are classified into initiating and killer types. Initiating type (caspase -2, -8, -9, -10 and -12), is triggered autocatalysis upon sensing death signals; and killer type (caspase-3, -6, and -7) is triggered proteolysis by initiating caspases [89]. Gürgen et al. [90] reported that when whey protein is used indiscriminately, and in the absence of sufficient physical activity, negative effects on the liver may occur, due to the increased apoptotic signs short-term, and increased inflammatory markers and hepatotoxicity long-term. They also reported increased levels of AST and ALT in whey protein-fed groups.

## 5. Conclusions

The aforementioned results show that whey protein concentrates can replace fish meal up to 27.7%, which gives the same growth performance, increases the total weight and the total return of the surviving fish after bacterial challenge, and improves gut health and immune status post-*A. hydrophila* challenge. However, high levels of whey protein concentrates are not recommended in fish diets, since they negatively affected the intestinal and liver tissues and increased the level of cellular apoptosis, as indicated by the increased caspase 3 activity. Further researches are recommended to evaluate the effect of fish meal replacement with WPC on the histopathological examination of the kidney and to test the capacity of serum IgM to clot the bacteria used for the challenge.

## Figures and Tables

**Table 1:** Proximate chemical composition of the experimental diets (g/kg).

Ingredients	WPC0	WPC13.8	WPC27.7	WPC41.6	WPC55.5
Fish meal	180	150	120	90	60
DWPC	-	25	50	75	100
SBM 44%	255	258	290	333	355
Ground corn	243	236	212	164.5	130
Corn gluten 60% cp	110	110	110	110	110
Wheat bran	90	100	90	90	100
Wheat	50	50	50	50	50
Fish oil	60	58	61	65	68
Dical. ph.	0	1	4.5	10	14
Methionine	0	-	0.5	0.5	1
Vitamin and mineral premix #	12	12	12	12	12
Chemical composition ##					
Crude Protein	336.2	335.8	335.6	336.1	336.5
Fat	94.6	90.8	91.4	92.8	93.5
Crude Fiber	37.4	38.5	38.9	40.7	42.5
NFE *	385.6	397.5	397.8	390.1	389.0
DE kcal/kg	2907.3	2905.1	2910.6	2904.1	2908.2
Lysine	18.3	17.8	17.9	18.4	18.3
Methionine	7.1	6.7	7.0	6.8	7.1
Calcium	10.4	9.2	8.9	8.7	8.9
AP **	9.1	8.7	8.8	8.9	8.8

* Nitrogen free extract. ** Available phosphorus. # premix: Each kilogram of premix contains: vitamin A 550,000 IU, vitamin D 110,000 IU, vitamin E 11,000 mg, vitamin K 484 mg, vitamin C 50 g, vitamin B1 440 mg, vitamin B2 660 mg, vitamin B3 13,200 mg, vitamin B5 1100 mg, vitamin B6 1045 mg, vitamin B9 55 mg, Choline 110,000 mg, Biotin 6.6 mg, Fe 6.6 g, Cu 330 mg, Mn 1320 mg, Zn 6.6 g, Se 44 mg, I 110 mg. ## According to NRC (2011).

**Table 2:** The effect of partial replacement of fish meal with whey protein concentrate on the growth performance and the whole body composition (g/kg) of Nile tilapia fingerlings.

Parameters	WPC0	WPC13.8	WPC27.7	WPC41.6	WPC55.5	Regression Analysis **	
						Linear	Quadratic
Growth performance parameters							
Initial wt. (g/fish)	18.62 ± 0.19	18.43 ± 0.04	18.71 ± 0.04	18.77 ± 0.07	18.73 ± 0.07	0.11	0.84
Final BW (g/fish)	30.08 ± 0.89	30.01 ± 0.79	28.62 ± 0.21	29.69 ± 1.16	28.61 ± 0.48	0.21	0.88
Daily BWG (g/fish)	0.16 ± 0.01	0.16 ± 0.01	0.14 ± 0.003	0.15 ± 0.01	0.14 ± 0.007	0.13	0.89
Total BWG (g/fish)	11.46 ± 0.74	11.57 ± 0.76	9.91 ± 0.25	10.92 ± 1.11	9.87 ± 0.55	0.13	0.89
Total FI (g/fish)	30.23 ± 1.64	27.40 ± 1.65	26.23 ± 1.01	30.62 ± 1.02	29.33 ± 0.60	0.72	0.09
FCR	2.67 ± 0.28	2.37 ± 0.06	2.65 ± 0.16	2.86 ± 0.32	2.98 ± 0.16	0.14	0.37
PER	1.14 ± 0.13	1.25 ± 0.03	1.13 ± 0.07	1.06 ± 0.11	1.001 ± 0.05	0.01	0.98
SGR (%/day)	0.68 ± 0.03	0.69 ± 0.03	0.60 ± 0.01	0.65 ± 0.05	0.60 ± 0.02	0.09	0.90
Survival% after bacterial challenge	66.66 ± 7.95	88.88 ± 2.63	91.11 ± 2.22	95.55 ± 2.47	97.77 ± 2.22	0.00	0.03
Total wt. of the surviving fish after bacterial challenge (g)	299.15 ± 27.89	409.81 ± 9.84	381.72 ± 10.17	425.74 ± 20.19	419.83 ± 15.02	0.001	0.03
Whole body composition (g/kg)							
Dry Matter #	222.6 ± 0.8	222.6 ± 8.7	229.0 ± 3.1	226.3 ± 3.5	230.1 ± 3.8	0.29	0.70
Crude protein *	652.8 ± 5.83	683.3 ± 21.73	661.0 ± 21.10	660.1 ± 11.3	683.3 ± 15.9	0.481	0.912
Fat *	156.6 ± 8.8	156.6 ± 3.3	170.0 ± 5.7	133.3 ± 8.8	136.6 ± 8.8	0.02	0.15
Ash *	113.0 ± 3.8	113.3 ± 8.4	100.06 ± 1.3	139.1 ± 0. 9	121.8 ± 2.5	0.01	0.31

** The regressions were considered significant when *p* ≤ 0.05. BW: Body weight; BWG: Body weight gain; FI: Feed intake; FCR: Feed conversion ratio; PER: Protein efficiency ratio; SGR: Specific growth rate. **#** On a fresh basis. * On dry matter basis.

**Table 3:** The effect of partial replacement of fish meal with whey protein concentrates on immune status, blood levels of growth hormone and nitric oxide, and kidney and liver function tests of Nile tilapia fingerlings at the end of the experiment.

Parameters	WPC0	WPC13.8	WPC27.7	WPC41.6	WPC55.5	Regression Analysis *	
						Linear	Quadratic
Immunological tests							
IgM (µg/mL)	237.33 ±2.02	251.00 ±6.80	256.66 ±10.03	255.66 ±12.12	260.33 ±12.12	0.120	0.501
Lysozymes (µg/mL)	14.89 ±0.29	18.48 ± 0.32	21.06 ± 3.32	22.22 ± 1.68	26.03 ±2.85	0.003	0.905
Complement 3 (µg/mL)	106.66 ±2.40	111.66 ±1.76	114.33 ±2.33	121.00 ±3.21	127.33 ±3.28	0.00	0.519
Phagocytic %	35.33 ±2.40	38.33 ± 2.02	52.66 ± 0.66	45.66 ± 1.20	42.66 ±1.76	0.002	0.00
Phagocytic index	1.25 ± 0.08	1.53 ± 0.14	2.98 ± 0.10	2.29 ± 0.10	1.84 ± 0.07	0.00	0.00
Growth hormone (ng/mL)	1.59 ± 0.05	2.04 ± 0.14	2.55 ± 0.28	3.03 ± 0.03	4.44 ± 0.29	0.00	0.02
NO (µmol/L)	55.34 ±2.24	61.34 ± 1.62	60.19 ± 2.86	65.94 ± 3.59	69.44 ±6.99	0.02	0.90
Liver function tests							
ALT (U/L)	12.03 ±0.11	11.03 ± 0.08	11.91 ± 0.02	12.80 ± 0.02	13.12 ±0.04	0.14	0.00
AST (U/L)	9.12 ± 0.04	7.90 ± 0.03	9.30 ± 0.02	9.73 ± 0.04	13.85 ±0.02	0.00	0.00
Kidney function tests							
Urea (mg/dL)	7.14 ± 0.06	6.92 ± 0.018	5.92 ± 0.03	13.86 ± 1.55	3.38 ± 0.06	0.00	0.001
Creatinine (mg/dL)	0.53 ±0.002	0.52 ± 0.002	0.53 ± 0.002	0.62 ± 0.004	0.56 ±0.001	0.002	0.07

* The regressions were considered significant when *p* ≤ 0.05.

**Table 4:** The effect of partial replacement of fish meal with whey protein concentrates on immune status, blood levels of growth hormone and nitric oxide, and kidney and liver function tests of Nile tilapia fingerlings after bacterial challenge test.

Parameters	WPC0	WPC13.8	WPC27.7	WPC41.6	WPC55.5	Regression Analysis *	
						Linear	Quadratic
Immunological tests							
IgM (µg/mL)	234.00 ±6.24	240.00 ±4.72	289.66 ±1.76	231.00 ±5.03	235.00 ±4.72	0.65	0.00
Lysozymes (µg/mL)	13.11 ±1.04	16.53 ± 0.28	26.18 ± 1.55	12.46 ± 0.96	16.22 ±0.13	0.49	0.00
Complement 3 (µg/mL)	103.66 ±3.17	113.33 ±2.33	141.33 ±4.97	103.00 ±2.51	106.00 ±4.58	0.63	0.00
Phagocytic %	50.16 ±2.48	59.33 ± 2.96	70.33 ± 2.02	64.66 ± 2.02	64.00 ±3.60	0.003	0.005
Phagocytic index	3.25 ± 0.05	3.34 ± 0.06	4.20 ± 0.04	3.73 ± 0.08	3.66 ± 0.07	0.00	0.00
Growth hormone (ng/mL)	1.57 ± 0.07	2.08 ± 0.40	9.97 ± 1.37	2.52 ± 0.35	2.02 ± 0.26	0.53	0.00
NO (µmol/L)	55.14 ±2.41	60.09 ± 0.64	89.11 ± 5.83	64.20 ± 3.61	62.97 ±2.93	0.53	0.00
Liver function tests							
ALT (U/L)	12.12 ±0.05	11.84 ± 0.06	10.10 ± 0.03	10.77 ± 0.03	10.35 ±0.03	0.00	0.00
AST (U/L)	7.52 ± 0.02	7.50 ± 0.01	7.31 ± 0.02	7.45 ± 0.03	8.01 ± 0.03	0.00	0.00
Kidney function tests							
Urea (mg/dL)	4.20 ± 0.03	5.50 ± 0.01	9.93 ± 0.24	4.82 ± 0.02	2.49 ± 0.02	0.00	0.00
Creatinine (mg/dL)	0.75 ±0.002	0.64 ± 0.004	0.66 ± 0.002	0.60 ± 0.003	0.64 ±0.003	0.00	0.00

* The regressions were considered significant when *p* ≤ 0.05.

**Table 5:** The effect of partial replacement of fish meal with whey protein concentrate on morphometric measures of the intestine (µm) of Nile tilapia fingerlings.

Parameters	WPC0	WPC13.8	WPC27.7	WPC41.6	WPC55.5	Regression Analysis *	
						Linear	Quadratic
At the experimental end							
Villus height	158.92 ± 12.28	174.47 ± 19.47	179.86 ± 16.11	237.86 ± 12.76	267.98 ± 37.05	0.001	0.323
Villus width	83.13 ± 5.57	92.01 ± 17.69	92.57 ± 8.68	69.13 ± 9.94	57.62 ± 9.59	0.047	0.133
Crypt depth	93.18 ± 6.41	68.01 ± 12.92	61.23 ± 6.04	58.15 ± 7.73	48.43 ± 6.12	0.001	0.277
Goblet cells	14.40 ± 1.43	15.20 ± 2.26	9.60 ± 0.67	16.00 ± 2.70	14.20 ± 2.05	0.94	0.36
Lymphocytic count **	193.20 ± 16.746	205.00 ± 7.328	216.00 ± 12.075	131.60 ± 19.816	55.20 ± 1.960	0.00	0.00
After bacterial challenge							
Villus height	188.97 ± 12.04	183.99 ± 21.56	165.01 ± 7.511	240.56 ± 10.65	270.50 ± 46.42	0.01	0.08
Villus width	72.21 ± 10.36	57.10 ± 2.96	129.88 ± 33.54	81.69 ± 16.99	94.60 ± 15.34	0.25	0.36
Crypt depth	56.63 ± 7.13	60.10 ± 10.21	49.40 ± 7.20	50.08 ± 10.48	37.38 ± 3.61	0.07	0.49
Goblet cells	54.00 ± 6.26	97.20 ± 8.35	7.80 ± 0.73	24.60 ± 1.46	12.40 ± 2.11	0.00	0.80
Lymphocytic count **	269.20 ± 20.901	242.20 ± 19.871	276.20 ± 23.155	289.20 ± 66.560	102.60 ± 11.303	0.01	0.01

* The regressions were considered significant when *p* ≤ 0.05. ** Lymphocytic count/field in high magnification 400x.

**Table 6:** The effect of partial replacement of fish meal with whey protein concentrates on the economic efficiency of the experimental diets of *Oreochromis niloticus* at the end of the experiment.

Parameters	WPC0	WPC13.8	WPC27.7	WPC41.6	WPC55.5	Regression Analysis *	
						Linear	Quadratic
Net profit (LE)	9.02 ±1.19	11.19 ±0.25	10.04 ±0.81	9.64 ±1.04	9.21 ±0.57	0.67	0.18
Total return (LE)	15.68 ± 1.13	18.01 ± 0.47	16.42 ± 0.86	16.66 ± 1.17	16.01 ± 0.66	0.81	0.25
Feed cost (LE)	4.66 ± 0.28	4.81 ± 0.11	4.37 ± 0.15	5.01 ± 0.09	4.80 ± 0.12	0.36	0.58
Total cost (LE)	6.66 ± 0.06	6.81 ± 0.28	6.37 ± 0.11	7.01 ± 0.15	6.80 ± 0.09	0.36	0.58
Feed cost (LE)/kg gain	45.73 ± 9.77	27.74 ± 0.73	36.09 ± 6.91	41.52 ± 10.03	41.97 ± 6.13	0.79	0.25
EE #	1.94 ± 0.28	2.34 ± 0.11	2.29 ± 0.17	1.91 ± 0.16	1.91 ± 0.08	0.41	0.12
PI % ##	11.77 ± 3.72	19.02 ± 0.87	14.41 ± 2.96	13.37 ± 3.33	11.73 ± 1.89	0.52	0.19
Total return after bacterial infection (LE) **	10.34 ± 0.91	16.01 ± 0.69	15.00 ± 1.13	15.95 ± 1.36	15.65 ± 0.53	0.005	0.01

* The regressions were considered significant when *p* ≤ 0.05. **#** Economic efficiency, ## Performance index, ** Total return of the total surviving fish after bacterial infection. LE: the currency of Egypt.
